# Use of Computational Models to Investigate Human Targets of Small Ubiquitous Molecules

**DOI:** 10.1002/cbic.70350

**Published:** 2026-04-24

**Authors:** Elena Frasnetti, Francesco Frigerio, Fabrizio Cinquini, Stefano A. Serapian, Silvia Pavoni, Giorgio Colombo

**Affiliations:** ^1^ Dipartimento di Chimica Università di Pavia Pavia Italy; ^2^ F&P Lab/A, R and D Eni SpA San Donato Milanese (Mi) Italy; ^3^ Upstream and Technical Services – TECS/STES – Eni Spa San Donato Milanese (Mi) Italy; ^4^ DOW‐R and D Eni SpA San Donato Milanese (Mi) Italy

## Abstract

The effects on human health of chemical compounds, which might be present in the environment due to natural or anthropogenic causes, are a fundamental aspect to be considered for the protection of public health and workers’ health and in the evaluation of industrial processes in terms of health protection and sustainability. Investigations focused on lesser known effects that have recently drawn more attention on potential human target proteins. Due to the complexity of biochemical interactions, it is not straightforward to determine the biological response resulting from exposure to a specific chemical. In this article, we tackle this issue by combining chemoinformatics tools and atomistic modeling to perform target identification for several compounds. The study was carried out using a publicly available database that collects relationships between chemicals, genes, and phenotypes and resulting diseases to validate the results of the target identification pipeline. Small molecules that may occur in occupational and nonoccupational settings were investigated. Finally, we discuss the potentialities and limitations of using these fast, computationally inexpensive methods in early‐stage target identification, both with and without performing a literature search for experimental data.

## Graphical abstract

Chemoinformatics and atomistic modeling were combined to identify protein targets of environmentally and occupationally relevant chemical compounds. A public database linking chemicals, genes, phenotypes, and diseases was used for validation. The study highlights the potential and limitations of these fast, low‐cost computational approaches for early‐stage toxicological target

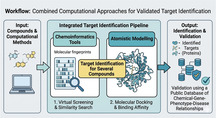

## 1 Introduction

A molecular understanding of the potential effects of chemicals on human proteins that oversee biochemical pathways relevant for human health is a key element in identifying potential issues also related to less known properties of chemical substances [1].

The interactions between small organic molecules and proteins are known to modulate protein function and consequently impact the activities of the signaling networks in which those proteins are involved [2, 3, 4, 5, 6, 7]. However, the targets for a large number of small molecules are unknown, limiting our capability to infer their impact on human cell biology.

This limitation in knowledge can be alleviated by the development of computational methods that are able to predict the potential targets of the chemicals identified. This methodology could be used in general to address the health effect of substances that are commonly found in the environment and that are produced in industrial environments to better understand the impact of specific activities. In this scenario, computational methods have already proven their potential in shedding light on the effect of compounds on human health [8, 9]. A recent published work of Rosa et al*.* [10], an explainable machine learning model trained on various toxicology databases, was used as a reference for the recognition of structural features that result in endocrine disruptive activity to carry out the present study. It should be noted that none of the substances that were investigated are classified for this effect at the moment of writing. Here, we describe the development of a novel computational pipeline for the identification and biological/pharmacological characterization of the potential interactions between small molecules and human cell receptors.

To develop an efficient approach to identify possible biochemical/pharmacological targets of the molecules/metabolites, we first select several molecules of interest because of their presence in the environment. Next, we define an integrated chemoinformatic and biological–structural approach to identify the possible preferential targets of the selected compounds. This step is performed by combining different approaches that define the biological targets of a certain chemical entity on the basis of its similarity with known ligands of that protein. We define a consensus of three different methods to highlight which molecules have a higher probability of binding a certain target.

Next, we use an atomistic docking approach to characterize the interactions between the selected/identified molecules and their putative targets and verify whether they reproduce the interactions experimentally observed with known ligands.

Finally, we compare the hypothesis formulated exclusively with in silico methods to experimental evidence, to evaluate the reliability of these simple and fast methods in identifying possible molecule–target pairs.

Although in theory this pipeline can be applied for any kind of compound, the goal in this article is the definition of the potential effect of specific molecules on human cell states by means of their interactions with specific receptors.

The potential health effect as endocrine disruptors (EDs), already addressed by the work of Rosa [10], of several hydrocarbons has been analyzed. Additionally, the same methodology has been used to try to have a better understanding of the interaction of substances that are perceived as “odorous” with smell receptors.

We suggest that this approach can be useful to identify interactions with human cell targets and reveal the origin of different types of effects. Additionally, this information can be relevant to define new starting materials for drug development, detoxification, and so forth.

## 2 Materials and Methods

### 2.1 Similarity Search Methods

The three similarity search methods employed in this study are the following:

• Similarity ensemble approach (SEA) [11].

The model first calculates a raw score, as the sum of Tanimoto scores for a query molecule calculated against many ligand sets, each associated with their own target. The score is then corrected with a statistical model of the expected similarity for random sets of ligands, to minimize the dependence of the score on the set size. Through the statistical model, it is possible to define *Z*‐scores and expectation values (*E* values) from the raw scores. In particular, the lower the *E* value obtained, the more reliable is the prediction of interaction between a query ligand and a specific target.

• SuperPred (SP) [12, 13, 14].

This method combines a similarity search (based on the Tanimoto score) with a physicochemical properties analysis. A well‐known metric to describe the main physicochemical features and define the drug likeliness of a molecule is Lipinski's rule of five: An orally active drug should not have more than 5 hydrogen‐bond donors, not more than 10 hydrogen‐bond acceptors, a molecular weight lower than 500 Da, and the octanol–water partition coefficient below 5. These parameters are calculated for both the query molecule and several molecules with a known ATC code (or anatomical therapeutic chemical; see WHO expert committee report https://www.who.int/publications/i/item/924120933X), a classification system that divides molecules into groups based on their chemical and pharmacological properties).

For every target in the output, both probability and model accuracy are reported, to evaluate the reliability of the prediction.

Finally, this model is trained exclusively on human proteins.

• SwissTargetPrediction (STP) [15, 16].

This method combines structural similarity (based on the Tanimoto score) and shape similarity [17]. The latter is calculated by first mapping every atom of a molecule to a 5D space, in which the first three dimensions describe the 3D conformation of the molecule, the fourth encodes atomic partial charges, and the fifth encodes atomic lipophilicity. Then, shape similarity is calculated on the basis of the distribution of the 5D distances of every atom of the molecule to six different centroid positions.

For each molecule, multiple conformations are generated, and all of them are compared to every conformation of another molecule: Only the highest similarity value is kept. Once both structural and shape similarity are calculated, they are combined using a multiple logistic regression as a final output.

For the predictions, this model only considers targets from *Homo sapiens*, *Mus musculus,* and *Rattus norvegicus* species.

### 2.2 Docking

The docking experiments were performed using the Glide software [18, 19] in Schrödinger‐Maestro (Schrödinger Release 2020‐4: Maestro, Schrödinger, LLC, New York, NY, 2020).

For each of the selected targets, one experimental structure was chosen (based on endogenous ligand and resolution of the structure) and downloaded from the Protein Data Bank. The protein was first prepared with the ‘Protein Preparation Wizard’ (Schrödinger Release 2020‐4: Maestro, Schrödinger, LLC, New York, NY, 2020), leaving the default options; if present, water molecules were removed. Both the experimental ligand and the identified binders were prepared using the LigPrep tool, to assign the correct protonation states in a pH interval between 6 and 8, using Epik. (Schrödinger Release 2020‐4: Maestro, Schrödinger, LLC, New York, NY, 2020.).

The docking experiments consisted of the following:

• Re‐docking of the experimental ligands inside the binding site. Root mean square deviation (RMSD) between the experimental and the docked pose was calculated (using the ‘Superimpose’ tool in Maestro), to estimate the comparability between the two poses. Additionally, this step was necessary to determine the docking score associated with the experimental ligand (i.e., docking score of the pose with lowest RMSD to the experimental pose).
• Docking of the suggested binders, to qualitatively compare the docking scores of such compounds to the one obtained by the reference experimental ligand.

In both types of experiments, the binding site was defined by selecting the endogenous ligand in the experimental structure, and a cubic grid (with a side size of 10 Å) centered on the ligand was created.

Finally, the standard precision scoring functions were employed, and a maximum of 15 output poses per ligand were generated.

## 3 Results and Discussion

### 3.1 Identification of Biological Targets: Molecular Similarity Search Analysis

The list of query molecules was selected according to publicly available literature [20]. The substances tested for potential ED effect were chosen among the nonclassified substances for which some literature observations were available [10] to investigate their potential interference/activity on human health processes. These substances chosen for their odorous effect were molecules selected either because of their ubiquitous presence in the environment or because they could be easily perceived due to their low odor threshold—that is, a threshold effect ranging from 0,07 ppb (for 3,3′‐thiodipropionic acid) to 10 ppm (for ethylbenzene).

To identify and predict possible targets, we used three different approaches that fall in the category of ligand‐based methods and, more specifically, in the similarity search domain. In this context, the similarity to known (endogenous, natural, or synthetic) ligands for which an experimentally solved 3D structure in complex with one or more proteins is publicly available is used to guide the identification of the targets.

**FIGURE 1:**
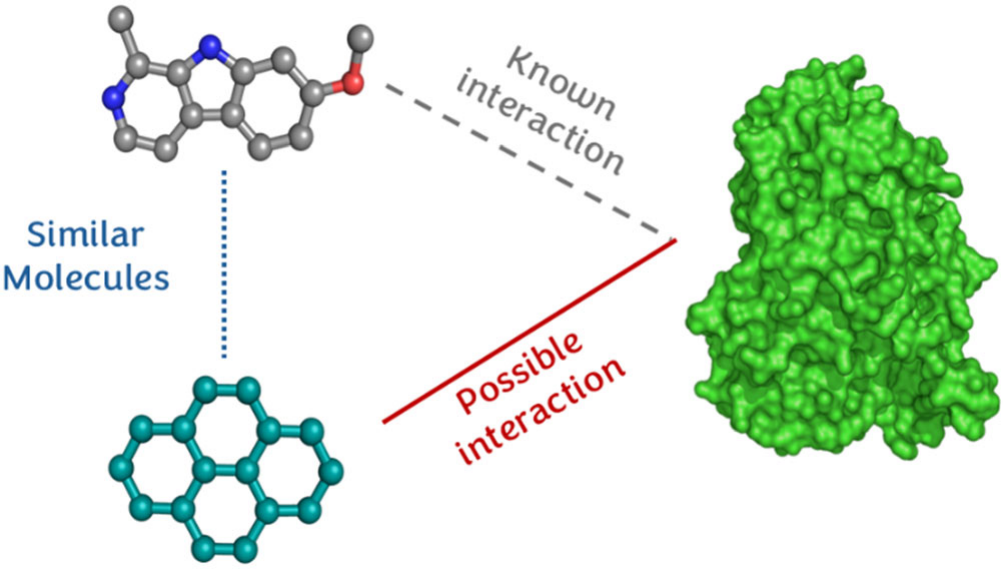
Schematic representation of the working hypothesis in similarity search methods. Given a known ligand–target pair, a second molecule (in teal) that shares high similarity with the known binder (in gray) might interact with the target.

The working hypothesis is that molecules with a similar structure and similar physicochemical properties tend to interact in a comparable way with the same targets (Figure 1). Therefore, by determining the similarity between a molecule and experimentally known binders, potential targets can be suggested for the query molecule.

Similarity among different molecules is evaluated on the basis of the Tanimoto coefficient, which ranges from 0 (very low/no similarity) to 1 (very high similarity). In this approach, which favors fast comparisons, molecules are first converted into fingerprints, that is, sequences of bits that encode the structural features of molecules [21]. The Tanimoto coefficient encodes the similarity between two sequences of fingerprints (corresponding to two molecules) in the following way:

$$T_{c}=\frac{C}{A+B-C}$$

where *A* is the number of bits set as 1 in the first fingerprint; *B* is the number of bits set as 1 in the second fingerprint, and *C* is the number of bits set as 1 in both fingerprints.

The models used to predict the potential target–binder pairs are SEA [11], SP, [12, 13, 14], and STP [15, 16].

Although all three methods are mainly based on the Tanimoto similarity between query compound and known binders, they differ from each other due to the inclusion of other physicochemical features in the definition of similarity, as well as the types of targets they have been trained on (please refer to Section 2 for details on each model).

Tables 1 and 2 summarize the results we obtained for the substances studied for their potential effect as ‘endocrine disruptors’ (EDs) and ‘odorous perception compounds’ (OCs), respectively. In particular, the tables report the number of human targets that were identified by each method as a potential target for the query molecule. Additionally, because SP only predicts human targets, the reported number in the table refers to the number of identified targets with a model accuracy ≥85% and a calculated probability of binding the query molecule ≥60%.

**TABLE 1:** Results for the similarity search analyses for the substances on which the endocrine disruption effect was investigated.

Compound	SEA	SP	STP
Naphthalene	9	35	3
Acenaphthylene	—	43	6
Perylene	6	49	3
Phenanthrene	32	48	3
Benzo(a)pyrene	8	54	—
Benzo(b)fluoranthene	4	68	3
Benzo(ghi)perylene	4	47	3
Benzophenone	104	34	12
Biphenyl	33	33	4
Cyclopenta(c,d)pyrene	1	57	6
Dibenzo(a,h)anthracene	25	56	3
Dibenzo(b, d)furan	11	41	1
Pyrene	4	44	3
Fluorene	14	46	2
Indeno(1,2,3‐cd)pyrene	5	67	—
n‐Hexane	24	44	3
Benzene	—	15	3
*o*‐Xylene	7	36	1
*m*‐Xylene	20	48	1
*p*‐Xylene	10	31	2
Ethylbenzene	28	38	3
Toluene	16	33	1
*o*‐Cresol	12	34	15

**TABLE 2:** Results for the similarity search analyses for the odorous compounds (OC).

Compound	SEA	SP	STP
1,2,3‐Trimethyl‐benzene	3	43	1
1,2,4‐Trimethyl‐benzene	5	49	1
1,3,5‐Trimethyl‐benzene	2	36	1
3‐3′‐Thiodipropionic acid	20	39	2
Acetaldehyde	1	17	—
Benzene	—	15	3
Butanoic acid	37	39	14
Cadaverine	17	53	15
D‐Limonene	—	42	4
Ethanol	—	24	—
Ethanolamine	1	31	—
Ethylbenzene	28	38	3
Hexanoic acid	104	53	33
Indole	7	48	17
*m*‐Xylene	20	48	1
Putrescine	11	42	10
*p*‐Xylene	10	31	2
Scatole	19	59	20
Triethylamine	—	30	—

Although these methods carry some differences, for both sets of molecules, it was possible to identify some common targets among all methods (SEA + SP + STP) or among pairs of methods. Tables 3 and 4 summarize how many common human targets between methods were found for the ED (first table) and OC (second table) set of molecules.

**TABLE 3:** Number of predicted targets common to different methods for the substances on which the endocrine disruptor (ED) effect was studied.

Compound	SEA + SP	SEA + STP	SP + STP
Benzo(a)pyrene	2	—	—
Benzo(b)fluoranthene	—	1	1
Benzo(g,h,i)perylene	1	1	1
Benzophenone	3	5	—
Biphenyl	1	—	—
Dibenzo(a,h)anthracene	3	2	1
Dibenzo(b,d)furan	—	1	—
Ethylbenzene	—	1	—
Fluorene	1	—	—
*m*‐Xylene	—	1	—
Naphthalene	—	1	—
n‐Hexane	4	—	—
*o*‐Cresol	—	2	1
*o*‐Xylene	—	1	—
Perylene	—	1	1
Phenanthrene	3	2	—
Pyrene	—	—	1
Toluene	1	1	—

**TABLE 4:** Number of predicted targets common to different methods for the odorous compounds (OC).

Compound	All methods	SEA + SP	SEA + STP	**SP** ** + STP**
3‐3′‐Thiodipropionic acid	—	—	1	—
Butanoic acid	—	—	5	—
Cadaverine	9	—	—	—
D‐Limonene	—	—	—	1
Ethylbenzene	—	2	—	—
Hexanoic acid	1	2	14	—
Indole	—	1	3	—
*m*‐Xylene	—	1	1	—
Putrescine	1	—	3	—
*p*‐Xylene	—	1	—	—
Scatole	1	—	1	1

## 4 Structure‐Based Filtering Step Using Docking Experiments

**FIGURE 2:**
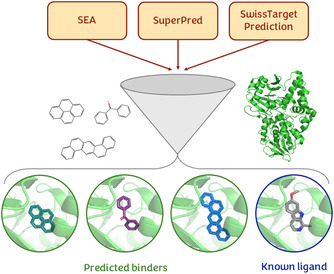
Filtering step with docking experiments. The common results among the similarity search methods are further investigated by performing docking experiments; the poses and scores of the predicted binders are compared to those obtained by an experimentally known ligand.

After identifying possible target–ligand complexes with the previous similarity search experiments, we selected the molecules predicted to bind a certain target from the consensus of at least two approaches for subsequent docking experiments into the binding site of the identified protein [22] (Figure 2). This approach is intended to evaluate whether the molecules can trace the main stereo‐electronic determinants for binding identified in experimentally known structures.

It is worth noting here that the results of a docking experiment are a set of possible poses of the ligand inside the binding site, where each pose is associated with a different docking score. This score—referred to as docking score—approximates the free energy of binding; therefore, the lower the docking score, the better the result. Furthermore, it is possible to calculate the ligand efficiency of a compound, which is the ratio between the docking score and the number of heavy atoms (i.e., all atoms beside hydrogens). This metric is particularly useful to compare the results of docking experiments for compounds that have different chemical structures and sizes.

The objective of this experiment is to have a qualitative result that can explain how favorable the binding between the query molecule and the predicted target is and how comparable the binding pose of the query molecule is to the one of a known binder.

Among the different pairs of predicted molecule target, we selected a subset for this type of experiment, following the criteria described below.

First, only complexes that were identified by at least two similarity search methods were considered. Furthermore, we chose only the targets for which an experimental structure with either an endogenous ligand or inhibitor was available (Table 5). In the case of multiple experimental structures, the one with the best resolution was generally chosen. The only exceptions included structures with a worse resolution but a more well‐known ligand (e.g., in the case of the GABA receptor, a structure in the presence of its endogenous ligand γ‐aminobutyric acid was selected in favor of other structures with other nonnatural binders but higher resolution).

**TABLE 5:** List of selected targets for the docking experiment. For each protein, the number of substances on which the endocrine disruptor effect was tested (second column) and odorous compounds (third column) that were identified as possible binders by the similarity search analysis.

Target	Substances tested for ED effect	Odorous compounds
Monoamine oxidase A	7	0
Hypoxanthine‐guanine phosphoribosyltransferase	6	0
Acetylcholinesterase	3	1
Aminopeptidase N	1	1
Carbonic anhydrase IV	0	2
Carbonic anhydrase XII	1	1
Cytochrome P450 1A2	2	0
Cytochrome P450 1B1	2	0
Geranylgeranyl pyrophosphate synthetase	1	1
Serotonin 2c (5‐HT2c) receptor	1	1
Cannabinoid CB2 receptor	0	1
Carbonic anhydrase VII	0	1
Carbonic anhydrase XIV	0	1
Cytochrome P450 2A6	0	1
Dopamine D1 receptor	1	0
Dual‐specificity phosphatase Cdc25B	1	0
Estrogen receptor alpha	1	0
Estrogen receptor beta	1	0
Fatty acid binding protein adipocyte	0	1
Fatty acid binding protein muscle	0	1
GABA‐B receptor	0	1
Histone lysine demethylase PHF8	0	1
HMG‐CoA reductase	0	1
Lysine‐specific demethylase 2A	0	1
Monoamine oxidase B	0	1
Peroxisome proliferator–activated receptor alpha	0	1
Peroxisome proliferator–activated receptor delta	0	1
Prostanoid EP2 receptor	0	1
Protein kinase C alpha	0	1
Quinone reductase 2	1	0
Serotonin 2b (5‐HT2b) receptor	0	1
Sphingosine 1‐phosphate receptor Edg‐3	0	1
Steroid 5‐alpha‐reductase 2	1	0

**FIGURE 3:**
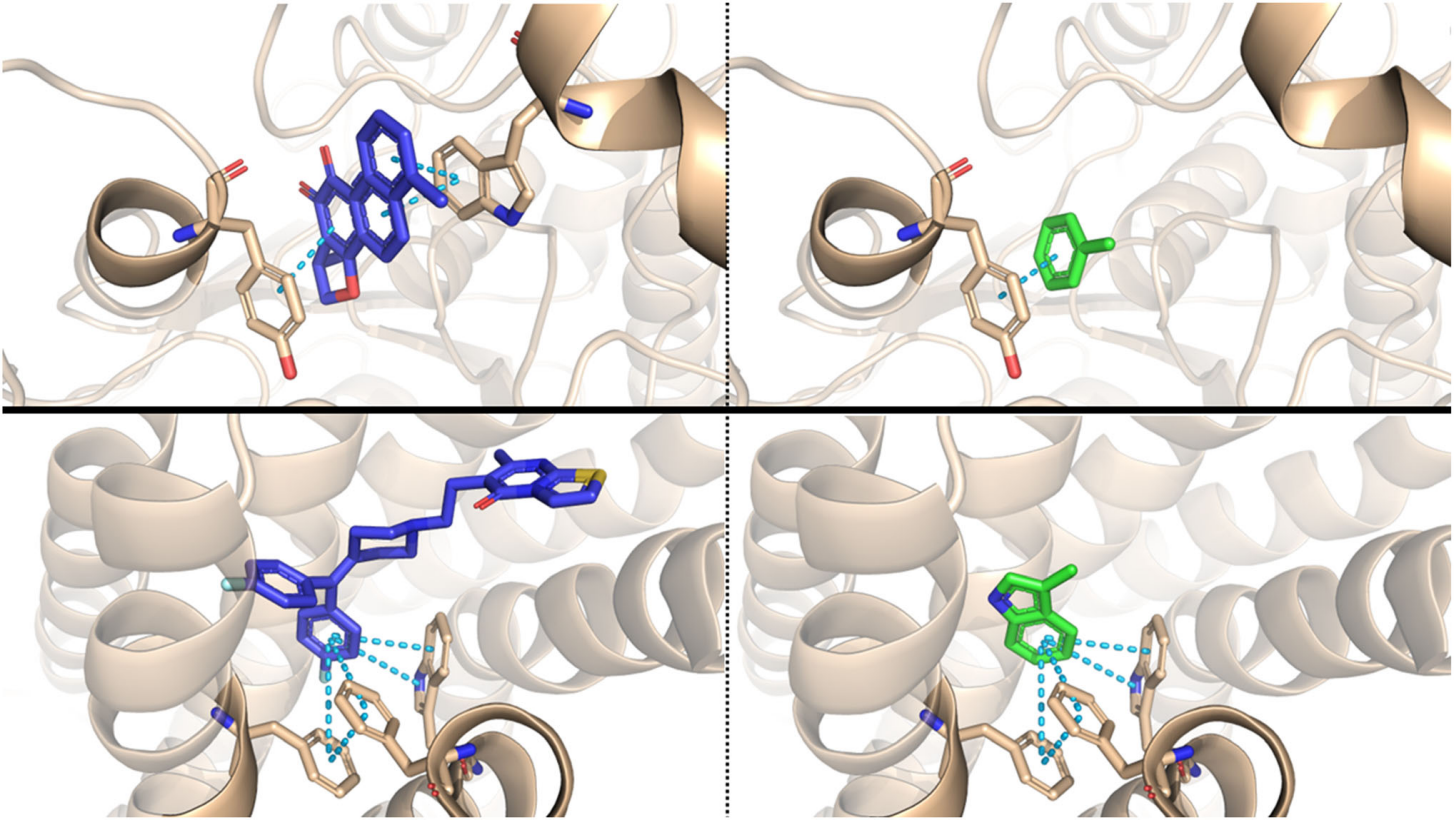
Comparison between docking poses of known binders (in blue, left) and query molecules (in green, right). Results on the top refer to the acetylcholinesterase (AChE) receptor (PDB: 4M0E), whereas results on the bottom refer to the Serotonin 2c (5‐HT2c) receptor (PDB: 6BQH).

In many cases, the query molecules obtained a higher ligand efficiency score compared to the experimentally bound ligands, suggesting that binding between the query molecules and their predicted targets may be favorable. Indeed, by comparing the docking poses of known ligands and query molecules, it is easily noticed that many of the fundamental interactions established in the experimental crystal structure are maintained by the potential binders (Figure 3).

Docking approaches have demonstrated the ability to rapidly generate viable models of complexes, which have often been validated experimentally and used proficiently in drug design campaigns.

Here, we set out to validate predicted models of interactions based on literature data. To progress toward this final goal, we made use of the Comparative Toxicogenomics Database (CTD) [23].

## 5 Validation of the Predictions with CTD

All the query molecules were searched on the CTD [23], which is a manually curated database that merges chemical–protein/gene interactions with disease–chemical/gene relationships.

For most molecules, it was possible to find the experimental validation for several targets predicted by either one or multiple similarity search methods (Table 6).

**TABLE 6:** Number of predicted targets validated by the CTD search. Additionally, if one validated target is predicted by multiple similarity search methods, the query molecule is written in bold. Substances studied for the ED effect are highlighted in orange, OC are highlighted in blue, and compounds that belong to both classes are highlighted in green.

	Similarity method		
Molecule	SEA	STP	SP
Benzo(a)pyrene	8	—	51
Benzo(b)fluoranthene	2	2	15
Benzo(g,h,i)perylene	2	—	1
Benzophenone	—	—	1
Dibenzo(a,h)anthracene	8	1	9
Dibenzo(b,d)furan	2	—	—
Indeno(1,2,3‐cd)pyrene	1	—	1
n‐Hexane	—	—	2
Naphthalene	1	1	4
*o*‐Cresol	—	3	—
Perylene	1	—	—
Phenanthrene	5	1	1
*p*‐Xylene	—	1	—
Pyrene	1	1	3
Toluene	1	1	4
Benzene	—	2	5
Ethylbenzene	—	1	1
*m*‐Xylene	—	—	1
1,2,4‐Trimethyl‐benzene	—	—	4
Acetaldehyde	—	—	2
Butanoic acid	2	1	5
D‐Limonene	—	—	2
Ethanol	—	—	16
Indole	1	1	—
Scatole	1	—	1

A particularly useful tool that is available on CTD is the so‐called CTD tetramer search [24], which allows to retrieve a chemical–gene–phenotype–disease relationship that is experimentally validated.

Through this tool, it is possible to validate the chemical–gene interaction and determine the phenotype/potential disease that could arise from this interaction.

For almost half of the query molecules (17 out of 38), it was possible to generate at least one tetramer, and for 13 of them, one of the predicted targets (from the similarity search analysis) was part of the tetramer (Table 7).

**TABLE 7:** Number of predicted targets found by the CTD tetramer search. Substances investigated for ED are highlighted in orange; OC are highlighted in blue, and compounds that belong to both classes are highlighted in green.

Molecule	Predicted targets in tetramers
Benzo(a)pyrene	21
Benzo(b)fluoranthene	2
Dibenzo(a, h)anthracene	4
Indeno(1,2,3‐cd)pyrene	1
Naphthalene	2
Phenanthrene	2
Pyrene	3
Toluene	1
Benzene	4
Ethylbenzene	1
Butanoic acid	2
D‐Limonene	1
Ethanol	3

Altogether, the results demonstrate the potentiality of our pipeline to relate binding to targets and their potential phenotypic effect.

Finally, we performed two case studies to determine whether through the pipeline it was possible to identify known genes associated with the endocrine disruptive activity or the odorous feature of some compounds, belonging to either class.

## 6 Investigating EDs: Case Study on Benzo(a)pyrene and n‐Hexane

Benzo(a)pyrene and n‐hexane were selected as representatives of the substances studied for the ED effect, to pinpoint interactions between genes related to the endocrine system and the query molecules.

The selection of such genes was performed according to the paper “Nuclear receptors are the major targets of endocrine disrupting chemicals” [25].

For both molecules, the selected genes were searched for in the corresponding CTD file, to determine whether the query molecule has been reported to have an effect on the aforementioned genes.

As Table 8 indicates, for benzo(a)pyrene it was possible to identify some experimental evidence of interactions with genes related to the endocrine system, suggesting that this compound might exhibit ED activity. Among them, four of them were previously predicted by the similarity search analysis (Table 9).

**TABLE 8:** Number of genes associated with the endocrine system that were found experimentally to be affected by either benzo(a)pyrene or n‐hexane.

Receptor Type	Tot genes	Benzo(a)pyrene	n‐Hexane
Estrogen	25	7	0
Androgen	5	1	0
Thyroid	17	6	0
Steroid metabolism	101	41	5
Retinoid X	13	5	0
Pregnane X	2	1	0
Peroxisome proliferator	5	2	0

**TABLE 9:** Genes associated with the endocrine system that were identified by the similarity search for benzo(a)pyrene.

Protein names	Gene Name	Type	Method
Nuclear receptor subfamily 1 group I member 2	NR1I2	Pregnane X	SP
Cytochrome P450 1B1	CYP1B1	Steroid	SEA
Nuclear receptor ROR‐beta	RORB	Steroid	SP
Thyroid hormone receptor alpha	THRA	Thyroid	SP

The substantial amount of experimental data available on CTD for this compound is potentially related to extensive studies regarding the toxicity of polycyclic aromatic hydrocarbons (PAHs) and their effect on human health. Indeed, the CTD tetramer file, which summarizes chemical–gene–phenotype–disease relationships, includes several diseases that might be caused by exposure to benzo(a)pyrene and PAHs in general, and some of them could be linked to the effect of this compound on the endocrine system itself.

As far as n‐hexane is concerned, only five genes, all belonging to the ‘Steroid metabolism’ category, were identified as interacting with this compound. However, none of these were predicted as potential targets by any of the similarity search models employed in this study.

## 7 Investigating Odorous Compounds: Case Study on D‐Limonene

D‐Limonene is a substance that can be found in natural products and commonly it is commonly used in fragrances, cosmetics, and cleaning agents. It has a typical orange‐like fragrance and an odor threshold of about 0.5 ppm. For these reasons, it was selected as a representative of the odorous compounds class and investigated further.

As in the previous case study, the selection of relevant genes was based on the paper "Mammalian olfactory receptors" [26].

Once again, the D‐Limonene CTD file was analyzed to find any of the selected genes that belong to the olfactory system, and the results are summarized in Table 10.

**TABLE 10:** Genes associated with the olfactory system that were found experimentally to be affected by D‐Limonene.

Type	Tot genes	D‐Limonene
Olfactory receptors	604	1
Vomeronasal receptors	18	0
Trace amine‐associated receptors	24	0
Formyl peptide receptors	13	0
Guanylyl cyclase GC‐D	1	0

D‐Limonene is reported to interact with the olfactory receptor OR2V1 [27], which is indeed annotated in the Limonene CTD file. On the other hand, none of the similarity search methods suggested this known interaction. This might depend on the types of targets that the different methods have been trained on. In fact, the similarity search method did not suggest any targets/genes that are related to the olfactory system. Therefore, these methods might be useful for identifying new potential interactions, rather than investigating the odorous feature of D‐limonene. On the other hand, the CTD might be more suitable if the goal is to explain phenotypes or relationships with disease (because it is possible to search for chemicals, genes, phenotypes, GO annotations, etc.).

## 8 Conclusions

The different similarity search methods are fast and simple tools for predicting new targets and could be employed in the early stages of target prediction workflows. However, the results vary on the basis of the targets considered when training the models. For instance, SP focuses exclusively on human targets, which explains its highest alignment with published data among the employed methods. Moreover, our findings are clearly open to and need to be experimentally validated in accordance with established standards.

Conversely, the CTD might be particularly useful when exploring the effect of exposure to a query molecule, as it supports searches based on chemicals, genes, phenotypes, or diseases. In particular, the CTD tetramer tool allows to link directly a query molecule with interacting genes, phenotypes, and the associated diseases. Furthermore, the database is manually curated and includes relevant data with literature references, but it relies heavily on the validity of experimental data. Because CTD is based on experimental data, one limitation is that if a molecule has not been extensively studied, the database may contain little or no information. In this scenario, similarity search methods, combined with other modeling techniques, such as docking experiments, may provide a better starting point for identifying potential targets for a given molecule.

In a more general context, the method described here could be used to generate a consensus in early drug‐discovery campaigns, for which indeed the three separate ligand‐pharmacology methods were originally developed. Consensus results on designed small‐molecule ligands could be used, on the one hand, to predict target engagement, whereas on the other hand to predict possible off‐target effects. We suggest that this aspect would be particularly worth exploring, as many interesting leads fail in advancements due to off‐target liabilities. Finally, it is tempting to suggest that further inclusion of flexibility and machine learning predictions of activities and binding modes [7, 28] could further complement and reinforce the general applicability of our approach to the pharmacological realm.

## Conflicts of Interest

The authors declare no conflicts of interest.

## Data Availability

The data that support the findings of this study are available from the corresponding author upon reasonable request.
